# A Kinetic Approach in the Evaluation of Radical-Scavenging Efficiency of Sinapic Acid and Its Derivatives

**DOI:** 10.3390/molecules22030375

**Published:** 2017-02-28

**Authors:** Neda Nićiforović, Tomaž Polak, Damjan Makuc, Nataša Poklar Ulrih, Helena Abramovič

**Affiliations:** 1 Biotechnical Faculty, University of Ljubljana, SI-1111 Ljubljana, Slovenia; neda.neca@hotmail.com (N.N.); tomaz.polak@bf.uni-lj.si (T.P.); natasa.poklar@bf.uni-lj.si (N.P.U.); 2 Slovenian NMR Centre, National Institute of Chemistry, SI-1001 Ljubljana, Slovenia; damjan.makuc@ki.si

**Keywords:** sinapic acid, antioxidant activity, radical scavenging kinetics, radical scavenging mechanisms

## Abstract

A kinetic approach was used to determine the radical scavenging activities of sinapic acid and its derivatives: sinapine, 4-vinylsyringol, syringic acid, syringaldehyde, and ethyl, propyl and butyl sinapate. The responses were expressed as rates of 2,2-diphenyl-1-picrylhydrazyl radical (DPPH˙) scavenging (*R*_S_), superoxide radical (O_2_˙^−^) scavenging (*R*_FF_), and β-carotene bleaching in the emulsion system (*R*_B_). For *R*_S_ and *R*_B_, the esters of sinapic acid showed the highest responses while, for *R*_FF_, this was seen for syringic acid. The effectiveness of the selected compounds for scavenging these free radicals was also determined at a fixed endpoint. The early response parameters were demonstrated to be good discriminators in assessing differences for antioxidants with comparable fixed endpoint activity. The primary feature that ranks the kinetic data and the endpoint determinations is interpreted in terms of the mechanisms of the reactions involved in each of the assays conducted.

## 1. Introduction

Phenolic compounds are considered to be great contributors to the health effects of plant-based diets mostly due to their ability to scavenge free radicals. For that reason they are extensively investigated for their antioxidant activity. Most of the in vitro methods for characterization of the radical scavenging properties of phenolic compounds concentrate on stoichiometric aspects at a fixed reaction time when steady-state is reached and, thus, they do not define the early responses of an antioxidant in a particular system [1]. Bearing in mind that free radicals such as hydroxyl (HO˙), hydroperoxyl (HOO˙) and lipid (RO(O)˙) radicals are short-lived in foods and tissues, long-term incubations can ignore the initial fast reactions, and instead give weight to the slower side reactions [2]. A significant aspect in the determination of antioxidant activity of phenolic compounds would also be the reactivity in terms of the response of an antioxidant at the early steps of the process [3], which is related to the kinetics of the free radical quenching [4] and is conditioned by the reaction mechanism.

The scavenging of free radicals by antioxidants can occur by two major mechanisms: hydrogen atom transfer (HAT) and electron transfer (ET). In addition, transitional mechanisms are also possible, which include proton-coupled electron transfer (PCET), electron transfer-proton transfer, (ET-PT), and sequential proton loss electron transfer (SPLET) [5,6,7]. According to Wright et al. [8] HAT and ET mechanisms must always occur in parallel, but with different rates. Which mechanism is going to be primary (i.e., with a higher rate) depends on the molecular properties of the antioxidant, the radical species involved, and the nature of the solvent, and while the end result is the same, the kinetics and the prospective for side reactions differ [5].

As far as the molecular properties of an antioxidant are concerned, thermodynamic parameters, such as bond dissociation energy (BDE), ionisation potential (IP), proton affinity (PA), and electron transfer enthalpy (ETE) are of specific importance in the determination of which mechanism is the favoured one in the scavenging of free radicals [9,10,11]. With the HAT mechanism, which involves quenching of free radicals by the donation of a hydrogen atom, the BDE of the O–H bond is a major factor for the scavenging activity, as it is related to the stability of the phenoxyl radical (ArO˙). In the ET mechanism, the IP is the most significant parameter in the evaluation of scavenging activity, as it implicates single electron transfer from an antioxidant to a free radical, and the stability of the thus-formed radical cation (ArOH˙^+^). The lower the IP, the easier the electron abstraction is. Extended delocalisation and conjugation of the π-electrons enhanced by resonance effects and planarity favour lower IP values and strongly influence the ability of phenolic antioxidants to donate a single electron [9]. The SPLET mechanism, a two-step process, includes deprotonation of an –OH group of a phenol, with formation of a phenoxide anion (ArO**^−^**), which can be characterised by the PA [12]. This then undergoes oxidation to ArO˙ as the second step of the reaction, which is related to the ETE [11]. These thermodynamics parameters are affected by the inductive effects of the substituents attached to the phenolic ring. BDE, IP, and ETE are lower with electron-donating groups (i.e., with positive inductive effects, +I), and increased with electron-withdrawing groups (i.e., with negative inductive effects, −I). The opposite effect can be noted in the case of the PA. Electron-withdrawing groups facilitate deprotonation of an –OH group, and electron-donating groups have the opposite effect [10].

In addition, the nature of the reaction medium also influences the mechanism through which the reaction would take place. For example, hydrogen bond accepting solvents (e.g., methanol, ethanol) strongly influence the rate of hydrogen abstraction from a phenolic compound, by decreasing it and, thus, favouring electron transfer processes [13].

**Figure 1:**
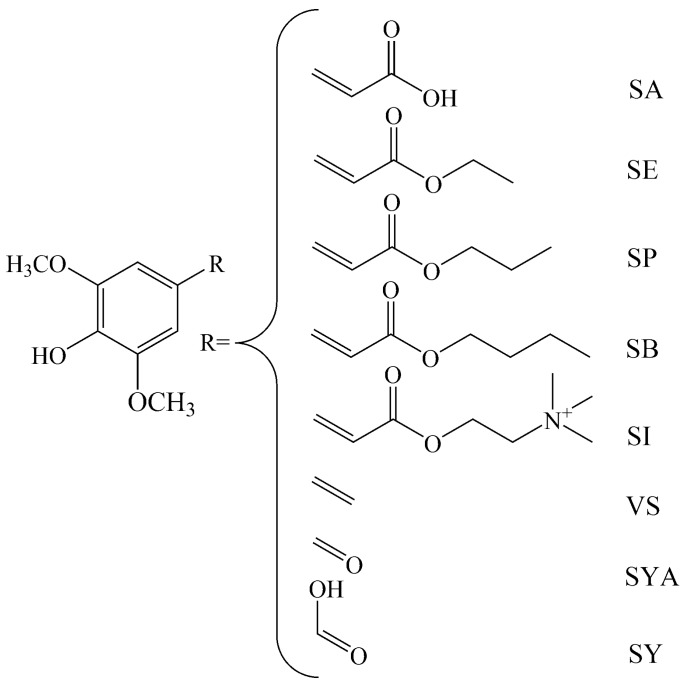
Structures of sinapic acid (SA) and its selected derivatives: ethyl sinapate (SE), propyl sinapate (SP), butyl sinapate (SB), sinapine (SI), 4-vinylsyringol (VS), syringaldehyde (SYA), and syringic acid (SY).

In recent years, sinapic acid (SA; i.e., 3,5-dimethoxy-4-hydroxycinnamic acid), and some of its derivatives, have been investigated because of their various biological activities, as reviewed by Nićiforović and Abramovič [14]. The aim of the present study was to investigate the radical-scavenging efficiency of SA and its derivatives: sinapine (SI; i.e., 3,5-dimethoxy-4-hydroxy-cinnamoyl choline), 4-vinylsyringol (VS; 3,5-dimethoxy-4-hydroxystyrene, also known as canolol), sinapoyl esters (e.g., ethyl [SE], propyl [SP], butyl [SB] sinapates), syringic acid (SY), and syringaldehyde (SYA; 4-hydroxy-3,5-dimethoxybenzaldehyde) (Figure 1) from the kinetics perspective. For this purpose, the scavenging activities of the selected phenolic compounds were analysed in terms of the 2,2-diphenyl-1-picrylhydrazyl (DPPH˙) radical, the superoxide radical (O_2_˙^−^), and lipid radicals generated in the β-carotene-linoleic acid emulsion system. The results obtained by this kinetics approach which provides the early rates of DPPH˙, O_2_˙^−^ and lipid radical scavenging for these selected compounds were compared to the conventional stoichiometry calculations obtained at a fixed endpoint. The ranking of these data is interpreted in terms of the reaction mechanisms of each assay, according to the structure and chemical properties of SA and its derivatives.

## 2. Results and Discussion

### 2.1. DPPH˙ Radical Scavenging Activity

**Figure 2:**
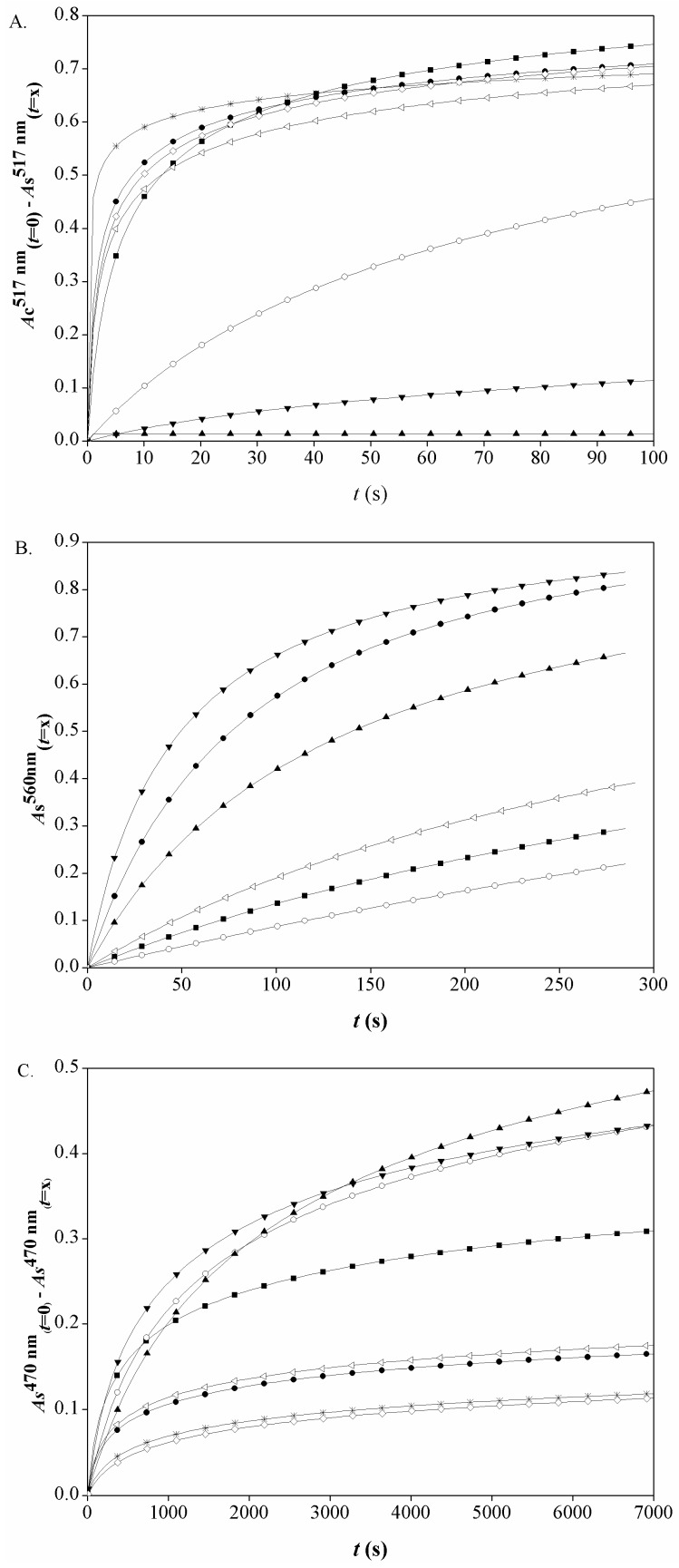
(**A**) Dependence of *A*c^517nm^_(*t*=0)_ − *A*s^517nm^_(*t*=*x*)_ on time of incubation at 6.67 × 10^−5^ mol/L of each tested compound; (**B**) dependence of *A*s^560nm^_(*t=x*)_ on time of incubation at 5 × 10^−3^ mol/L of each tested compound; and (**C**) dependence of *A*s^470nm^_(*t*=0)_ − *A*s^470nm^_(*t=x*)_ on time of incubation at 2 × 10^−4^ mol/L of each tested compound. (■), sinapic acid—SA; (○), syringic acid—SY; (▲), syringaldehyde—SYA; (●), ethyl sinapate—SE; (∗), propyl sinapate—SP; (◊), butyl sinapate—SB; (◁), 4-vinylsyringol—VS; (▼), and sinapine—SI.

DPPH˙ radical undergoes a colour change from purple to yellow upon receiving an electron or hydrogen from an antioxidant, and the effects of the investigated antioxidant can be estimated by measurement of the decrease in its absorbance at 517 nm. To determine the early responses of the selected antioxidants towards DPPH˙ radicals, the change in absorbance at 517 nm (*A*c^517nm^_(*t*=0)_ − *A*s^517nm^_(*t*=*x*)_) was plotted as a function of time (Figure 2A). The value *A*c^517nm^_(*t*=0)_ − *A*s^517nm^_(*t*=*x*)_ refers to the content of the DPPH˙ radical scavenged at *t = x*. The dependence of *A*c^517nm^_(*t*=0)_ − *A*s^517nm^_(*t*=*x*)_ on *t* was quantified for the first 100 s of each incubation, and is described by the power function given in Equation (1):

(1) $$y=1-\frac{1}{{\left(1+a\cdot t\right)}^{b}}$$

where *y* is *A*c^517nm^_(*t*=0)_ − *A*s^517nm^_(*t*=*x*)_.

The curves in Figure 2 were plotted on the basis of parameters a and b of Equation (1), which were obtained by non-linear regression analysis. The corresponding determination coefficients (*r*^2^) are presented in Table 1. The kinetics of the scavenging DPPH˙ radicals were quantified by the estimation of the rate of DPPH˙ scavenging (*R*_S_), as the first derivative of the power function given in Equation (2), with the results from these data given in Table 1.

(2) $$R=\frac{ab}{{\left(1+a\cdot t\right)}^{b+1}}$$

The same principle was applied for the kinetics of the scavenging of O_2_˙^−^ and lipid radicals by the investigated SA derivatives.

As the rate of radical scavenging is related to the slope of the curve at a specific time point, and as can be seen, the highest response occurs within the first few seconds, *R*_S_ was calculated at *t* = 0.1 s. Among all of the investigated compounds, the highest responses for scavenging of the DPPH˙ radicals were shown by the sinapate esters (SE, SP, SB) and VS, which caused sharp rise in *A*c^517nm^_(*t*=0)_ − *A*s^517nm^_(*t*=*x*)_ in the first 5 s of incubation (Figure 2A). This was especially the case for SP, where the increase in *A*c^517nm^_(*t*=0)_ − *A*s^517nm^_(*t*=*x*)_ occurred almost instantly, after which the reaction slowed down. In a study on the kinetics of DPPH˙ scavenging, Foti et al. [13] showed, on the example of methyl sinapate, that the introduction of alkyl moiety increases the reactivity of SA, which is in agreement with our results. On the other hand, for SA and SY, the increase in *A*c^517nm^_(*t*=0)_ − *A*s^517nm^_(*t*=*x*)_ was more gradual.

**Figure 3:**
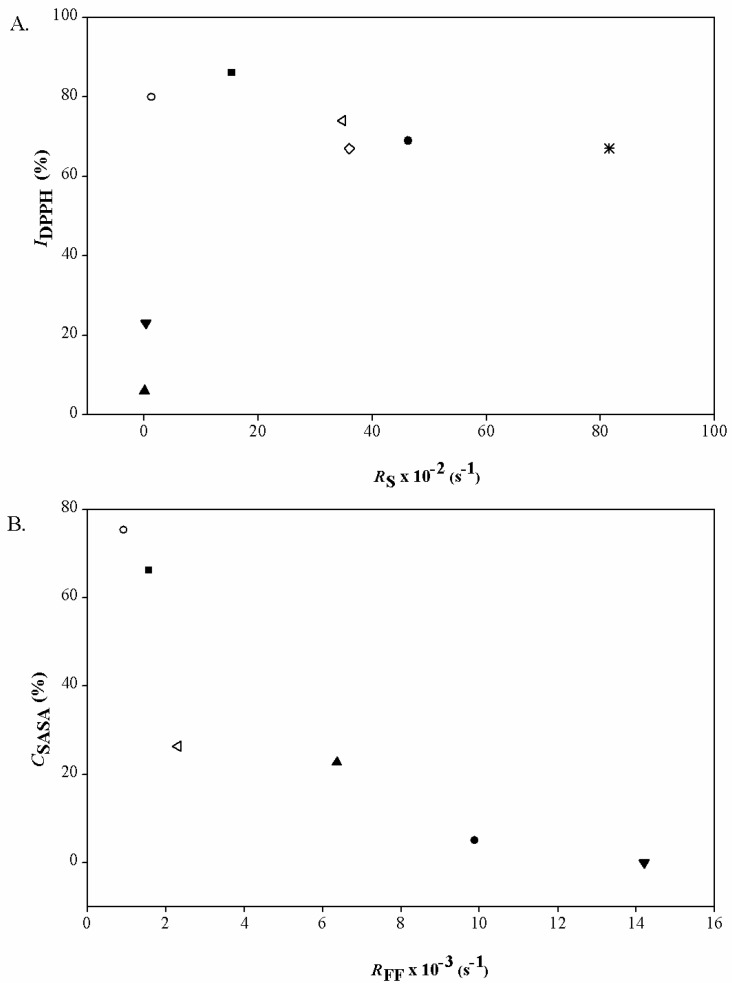
(**A**) Dependence of the 30-min endpoint for the DPPH˙ scavenging activity (*I*_DPPH_) on rate of DPPH˙ radical scavenging (*R*_S_) at 6.67 × 10^−5^ mol/L of each tested compound; (**B**) dependence of the superoxide radical scavenging activity coefficient (*C*_SASA_) on rate of formazan formation (*R*_FF_) at 5 × 10^−3^ mol/L of each tested compound. (■), sinapic acid—SA; (○), syringic acid—SY; (▲), syringaldehyde—SYA; (●), ethyl sinapate—SE; (∗), propyl sinapate—SP; (◊), butyl sinapate—SB; (◁), 4-vinylsyringol—VS; (▼), and sinapine—SI.

The amount of DPPH˙ scavenged by the same concentration of the selected compounds as used in the kinetics study was also determined at the fixed endpoint at 30 min, and expressed as percentage of scavenged DPPH˙ (Table 1, *I*_DPPH_). No correlation was found between *R*_S_ and *I*_DPPH_ (Figure 3A). When the fixed endpoint was reached, the order of activity was clearly different, with SA and SY scavenging most of the DPPH˙ radicals. The same tendency for the data obtained at a fixed endpoint for SA and its esters were also reported by Teixeira et al. [15].

The generally accepted mechanism of oxidation of phenols in a reaction with DPPH˙ in protic solvents, such as methanol or ethanol, is considered as SPLET based. Slower response to DPPH˙ at the early step of the reaction for SA than seen for its esters is ascribed to the suppressive influence of the carboxylate group (–COO^−^) on the deprotonation of ArOH (Equation (3)). By its electron-donating effects, –COO^−^ increases PA, which decreases *R*_S_ of SA versus its esters, where the deprotonation of ArOH is supported by the electron-withdrawing effect of the ester moiety (–COOR). This is in accordance with Foti et al. [13]. This suppressive influence of –COO^−^ on the rate of DPPH˙ scavenging was reported by Ordoudi et al. [16], who observed notably higher kinetics determined in ethanol for catechol than for its derivative bearing the –COO^−^ group, protocatechuic acid. On the other hand, at the second step of the process (Equation (4)), where the ETE is the major influencing factor, the effects of the –COO^−^ and –COOR functional groups are opposite. The electron transfer from ArO^−^ to DPPH˙ of esters is surpassed by SA, as it is supported by the +I of –COO^−^, which stabilises ArO˙ and results in greater activity (i.e., higher *I*_DPPH_) of SA than its esters at the fixed endpoint. Based on the PA and ETE given by Chen et al. [11], which were obtained using density functional theory calculations, in the example of ferulic acid and its ethyl ester, where lower PA and higher ETE were calculated for ferulic acid than for ethyl ferulate, it can be expected that SA has a better early response and its ester SE has a greater fixed endpoint DPPH˙ scavenging activity. However, our experimental data, as shown above, indicates the importance of considering the ionic forms of these phenolic acids in polar solvents under pH conditions above p*K*a values for their –COOH group.

(3) $$\mathrm{ArOH}⇆{\mathrm{ArO}}^{-}+H^{+}$$

(4) $${\mathrm{ArO}}^{-}+{\mathrm{DPPH}}^{˙}\to {\mathrm{ArO}}^{˙}+{\mathrm{DPPH}}^{-}$$

(5) $${\mathrm{DPPH}}^{-}+H^{+}\to {\mathrm{DPPH}}_{2}$$

(6) $${\mathrm{ArO}}^{˙}+{\mathrm{DPPH}}^{˙}\to \mathrm{products}$$

(7) $${\mathrm{ArO}}^{˙}+{\mathrm{ArO}}^{˙}\to \mathrm{dimer}$$

Sinapic acid esters showed the highest *R*s of all of the tested compounds, while at the fixed endpoint, their activities were moderate where the presence of an electron-withdrawing –COOR group destabilises their ArO˙, which leads to a possible reversible reaction with DPPH˙ (Equation (4)). This was shown in a study by Foti and Daquino [17], and was confirmed by the flat curves that followed the initial rapid increase in *A*c^517nm^_(*t*=0)_ − *A*s^517nm^_(*t*=*x*)_ (Figure 2A). On the other hand, the ArO˙ of SA and SY might be involved in the subsequent slower reactions before equilibrium is reached, which results in a high *I*_DPPH_. Namely, as well as the previously-described key reactions of SPLET (Equations (3) and (4)), ArO˙ can undergo the latter slower process to the secondary reactions of the phenol-derived radicals with the remaining DPPH˙, as well as to further chemical reactions among ArO˙ themselves, such as polymerisation or disproportionation (Equations (6) and (7)) [15]. Indeed, it is known that hydroxycinnamic acids can undergo oxidative cross-coupling, which leads to the formation of dimers, some of which have radical scavenging activities themselves [18]. Considering the ranking of the ester early responses towards the DPPH˙ radicals in relation to the alkyl chain length in the –COOR, steric accessibility to the hindered reaction site in DPPH˙ radical has been recognized as an important factor that limits the reaction rate [19,20], and that probably results in the lower *R*s of SB than observed for SE and SP.

The absence of an unsaturated ethylenic side chain and the closeness of the –COO^−^ group to the –OH group in the molecule of SY increase its suppressive effects on the deprotonation of ArOH, which results in the particularly lowered early rate of the reaction of SY in comparison to SA. This is in accordance to data reported by Ordoudi et al. [16] and Terpinc et al. [4], where the insertion of an ethylenic chain and the formation of a hydroxycinnamic derivative, caffeic acid, resulted in a profound increase in the reaction kinetics as compared to protocatechuic acid. The stabilizing effect of –COO^−^ on ArO˙ due to its proximity to the –OH in SY would be expected to lead to higher activity of SY than SA at the fixed endpoint. However, it appears that this phenomenon in SY is overcome by the absence of the ethylenic side chain, which leads to lower resonance delocalisation (i.e., lower stabilisation of ArO˙), and resulted in a slightly lower antioxidant activity for SY than for SA at the fixed endpoint (i.e., lower *I*_DPPH_).

The elimination of the –COO^−^ group in SA results in the formation of VS. The vinyl group in the molecule of VS has high electron density (+I effect), which would also have similar effects to –COO^−^ for deprotonation of ArOH and stabilisation of ArO˙, but would be less effective. This resulted in lower *R*s for VS compared to *R*s of the esters, although it remained higher than *R*s of SA and SY. At the fixed endpoint, the activity of VS with the more stabilised ArO˙ was higher than that of the esters, but lower than for SA and SY, which reveals a less established effect of the vinyl group than –COO^−^. SYA showed low activity in scavenging DPPH˙ radicals. The strong electron-withdrawing influence of –CHO in the molecule of SYA decreases its overall activity by destabilising ArO˙.

The interrelationship between *R*s and *I*_DPPH_ confirms that the *R*s is more affected by the nature of *p*-substituent on the formation of ArO^−^, while *I*_DPPH_ is dependent on the stability of ArO˙ and possible secondary reactions. In Figure 3A. it can be seen that *R*s is more sensitive to the differences in inductive effect of the *p*-substituent since its values significantly differ between compounds with comparable *I*_DPPH_. This suggests that *R*s is a better discriminator than *I*_DPPH_ in characterisation of antioxidants with similar activity at the fixed endpoint, such as in the case of SE, SP, SB and VS or SA and SY.

### 2.2. Superoxide Anion Scavenging Activity

Superoxide anion radicals are continuously produced in aerobic cells [21], and although they are not a highly reactive short-lived free radical species, their over-production can lead to cell damage and mutagenesis [22]. In the present study, O_2_˙^−^ were generated in the aerobic phenazine methosulphate (PMS)—β-nicotinamide adenine dinucleotide (NADH) system and monitored by following the reduction of nitroblue tetrazolium (NBT) (i.e., blue formazan formation). In the presence of an antioxidant, the reduction in NBT is suppressed due to the O_2_˙^−^ radical scavenging activity of the antioxidant [23]. To follow the kinetics of O_2_˙^−^ radical scavenging by the selected SA derivatives, the changes in absorbance at 560 nm were monitored for 300 s. The data are plotted as the dependence of *A*s^560nm^_(*t=x*)_ on *t*, where *A*s^560nm^_(*t=x*)_ represents the content of formazan at *t = x* (Figure 2B.). A slower increase in *A*s^560nm^_(*t=x*)_ denotes more suppressed formazan formation (whereby O_2_˙^−^ is more successfully scavenged). The kinetics of O_2_˙^−^ scavenging of the selected compounds were determined by calculation of the rates of formazan formation (*R*_FF_), through the power function model presented in Equation (1), where *y* denotes *A*s^560nm^_(*t=x*)_ (the corresponding parameters a and b were obtained by non-linear regression), and with the use of Equation (2) at *t* = 10 s (Table 1). The activities of the investigated compounds were also determined at the fixed endpoint, and these are expressed as the coefficient of O_2_˙^−^ radical scavenging activity (*C*_SASA_), calculated as in Terpinc and Abramovič [4].

Contrary to the DPPH˙ scavenging assay, the O_2_˙^−^ scavenging assay is a competition assay, where the antioxidant (i.e., the SA derivatives) and detector (i.e., NBT) compete for the radical reactive species (O_2_˙^−^), as stressed by Balk et al. [24]. This is not the case for the DPPH˙ scavenging assay, because DPPH˙ is both a radical and a detector [5]. Therefore, for the O_2_˙^−^ scavenging assay, the shapes of the curves cannot be interpreted in the same way as for the DPPH assay, and lower *R*_FF_ indicates better response of an antioxidant for the scavenging of O_2_˙^−^ radicals. SY and SA showed the greatest reactivities towards O_2_˙^−^ radicals among these tested compounds (Table 1). It appears that any modification to the side chain in the *p*-position to an –OH group in the selected SA derivatives resulted in lower O_2_˙^−^ radical scavenging. This activity was indeed minor for SE. Due to the low solubilities of SP and SB, these *R*_FF_ were not determined. The interrelationships between *R*_FF_ and *C*_SASA_ (Figure 3B) show good agreement between the determination of O_2_˙^−^ scavenging activity of these antioxidants at the early stage and at the fixed point of the reaction (i.e., at 5 min). However, in the same way as in the DPPH˙ scavenging assay, a kinetic perspective offers the possibility to find a distinction between two compounds which show corresponding activities at the fixed endpoint, as in the case of VS and SYA. This again shows the greater influence of the nature of the *p*-substituent on the early response than on the endpoint activity of O_2_˙^−^ scavenging.

The O_2_˙^−^ scavenging assay has not been classified into any of the mechanistic models mentioned in the literature. The order of activity here suggests that the reactivity of the investigated compounds towards O_2_˙^−^ scavenging does not depend on the acid-base equilibrium of the –OH group, which was seen for the DPPH˙ scavenging. In addition, as noted by Halliwell and Gutteridge [25], in aqueous solutions, the proton affinity of O_2_˙^−^ is low, as its charge density is decreased due to solvation. On this basis, it is reasonable to exclude SPLET as the mechanism of O_2_˙^−^ quenching in an aqueous environment. The compounds with the greatest activities are those that contained groups with electron-donating properties (e.g., SA, SY, VS), which lowered both BDE and IP and, thus, both of the HAT and ET mechanisms are considered for discussion. However, as explained by Nenadis and Siskos [12], the BDE is affected by local electronic phenomena and not by the whole structure of the compound, while on the other hand, the whole structures influence the IP. There is considerable difference in the reactivities between compounds with electron-donating and electron-withdrawing groups in the *p*-position to –OH. The +I effect of –COO^−^ of SY and SA, and of the vinyl group in VS, increases their reactivities towards O_2_˙^−^ by decreasing the IP of the –OH group and stabilising ArOH˙^+^ (Equation (8)), as opposed to the −I effect of –CHO in SYA and of –COOR in SE. Bearing in mind, as previously noted, that hydrogen bond accepting solvents (in this case the aqueous environment) favour electron transfer processes, it might be expected that O_2_˙^−^ scavenging would occur through ET mechanism, and the data in the present study support this. Although in this mechanism electron transfer is followed by deprotonation of the radical cation, which leads to the corresponding ArO˙ (Equation (9)), which would be hindered by electron donating groups (e.g., –COO^−^ in SY and SA), the rate-determining step is the electron donation [12]. In agreement with this, no influence of the second step (Equation (9)) on the *R*_FF_ was noted in the present study.

(8) $$\mathrm{ArOH}+O_{2}^{˙-}\to {\mathrm{ArOH}}^{˙+}+O_{2}^{2-}$$

(9) $${\mathrm{ArOH}}^{˙+}⇆{\mathrm{ArO}}^{˙}+H^{+}$$

### 2.3. Lipid Radical Scavenging Activity

Lipid peroxidation is a crucial step in the pathogenesis of several diseases, as well as a major mechanism that can lead to deterioration of food quality. This process involves the generation of different lipid radical species, which include the highly reactive peroxyl (ROO˙) and alkoxyl (RO˙) radicals [26]. The lipid radical scavenging activities of the investigated SA derivatives were estimated using a β-carotene bleaching assay in a linoleic acid emulsion system. Linoleic acid oxidation is catalysed by heat, and the lipid radicals that are formed react with β-carotene to form a stable β-carotene radical; this results in decreased absorbance at 470 nm. Figure 2C. shows the relationship between *A*s^470nm^_(*t=*0)_ – *A*s^470nm^_(*t=x*)_ and time, where *A*s^470nm^_(*t=*0)_ – *A*s^470nm^_(*t=x*)_ refers to the β-carotene bleached at *t* = *x*. The rate of β-carotene bleaching (*R*_B_) was calculated as for the previous two methods, with *y* in Equation (1) denoting *A*s^470nm^_(*t=*0)_ – *A*s^470nm^_(*t=x*)_ (Table 1). A stronger response in lipid radical scavenging in the linoleic acid emulsion system is seen by lower *R*_B_. The lipid-radical scavenging efficiencies were also determined at a fixed endpoint (60 min), and are expressed as the antioxidant activity coefficients (*C*_AA_) [4].

**Figure 4:**
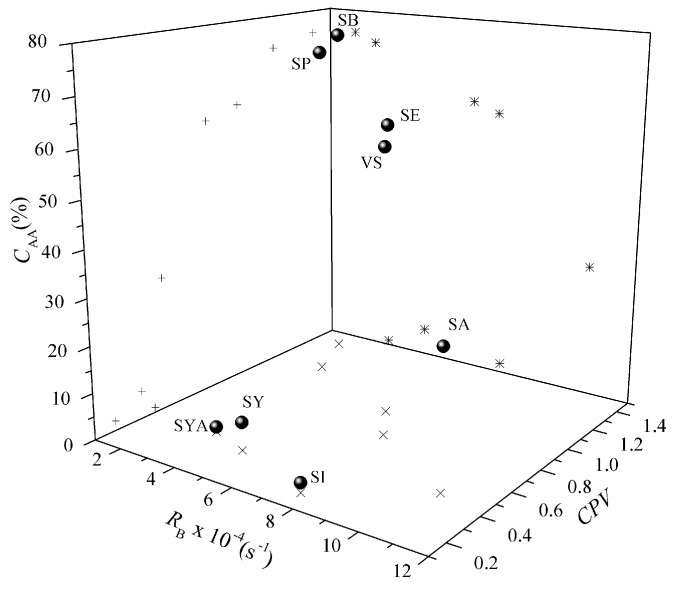
Interrelationship of the rate of β-carotene bleaching (*R*_B_), antioxidant activity coefficient (*C*_AA_) and chromatographic partition value (*CPV*) for the tested compounds (●), with projections of individual correlations (∗), *R*_B_ vs. *C*_AA_; (×), *R*_B_ vs. *CPV*; (+), *C*_AA_ vs. *CPV.* Sinapic acid (SA), syringic acid (SY), syringaldehyde (SYA), ethyl sinapate (SE), propyl sinapate (SP), butyl sinapate (SB), 4-vinylsyringol (VS), and sinapine (SI).

Whether we consider a food or a biological antioxidant, a notable factor that affects its efficacy and function is its partitioning properties between the lipid and aqueous phases [27]. Due to their hydrophilic nature, hydroxycinnamic acids cannot be used in oil-based environment, which is an important issue in industrial applications, or to be effective as antioxidants in biological systems. Various studies have shown that antioxidants with higher lipophilicity show improved activities in emulsion systems due to their better partitioning properties [28,29]. This allows such antioxidants to be positioned at the water-lipid interface and, thus, to prevent any initial reactions between aqueous radicals and lipids. Nevertheless, the aim here of the present study was to investigate whether the same principle can be applied to the early response of an antioxidant towards lipid radicals (ROO˙, RO˙) in an oil-in-water emulsion system. The interrelationship between *R*_B_, *C*_AA_ and chromatographic partition values (*CPV*) is given in Figure 4. The majority of the investigated compounds that showed good early responses towards lipid radicals also showed good scavenging activities at the 60 min endpoint, except for SYA, SY, and SI, where the *R*_B_ values do not commensurate with the *C*_AA_ values. Among the selected compounds, SB together with SP showed the strongest early response towards lipid radical scavenging. These two compounds also showed the highest *C*_AA_, and the highest lipophilicity. It can be seen that the early response is less dependent on lipophilicity, while *C*_AA_ is well correlated with the *CPV*.

The reaction of such phenolic compounds with lipid radicals is noted as the HAT mechanism [5]. RO(O)˙ radicals are highly reactive, and the reaction (Equation (10)) is essentially irreversible [30,31]. Therefore, considering the mechanism, as the inhibition of lipid peroxidation by an antioxidant is usually correlated to the BDE, it would be expected that the reactivity towards lipid radicals in heterogeneous systems, such as emulsions, should be increased by functional groups that stabilise ArO˙, such as the –COO^−^ in the molecules of SY and SA. However, in these systems, the distribution of the antioxidant between the oil, interfacial, and aqueous regions becomes a major factor for its activity. Esterification of SA clearly leads to increased lipophilicity, and to both greater early responses and activities at the fixed endpoint, despite the mildly destabilising −I effect of –COOR on the stability of their ArO˙. In addition, as noted by Burton and Ingold [32], the ArO˙ formed according to Equation (10) eventually reacts with a second radical (Equation (11)), and the molecular products thereby formed move the reaction forward. Although SYA, SY, and SI might be reactive towards lipid radicals and be able to scavenge those that are distributed on the surface of lipid droplets, thus showing a moderate early response, they will not distribute themselves within the lipid–water interface resulting in low *C*_AA_. In Figure 4 it can be seen that, again, the kinetic parameter *R*_B_ can assist in differentiation between two compounds with similar *C*_AA_, as in the cases of SB and SP or SE and VS.

(10) ArOH+RO(O)˙→ArO˙+RO(O)H

(11) $${\mathrm{ArO}}^{˙}+\mathrm{RO}{\left(O\right)}^{˙}\to \mathrm{molecular}\;\mathrm{products}$$

The reports on the antioxidant activity of SI in the literature are somewhat contradictory [33,34,35,36], while the data from the present study show little or no activities in any of the assays carried out. Esterification of SA with choline evidently reduces its radical-scavenging ability, as introduction of the positive-charged choline contributes to destabilisation of the corresponding ArO˙, which prevents it from donating a hydrogen atom or electron to the free radical species.

## 3. Methods

### 3.1. Materials and Reagents

Chloroform and ethanol (96%) were from Merck (Darmstadt, Germany). SA, SY, SYA, β-carotene, DPPH˙ reagent, linoleic acid (95%), Tween 20, NBT, NADH, and PMS were from Sigma (Sigma-Aldrich GmbH, Taufkirchen, Germany). Potassium dihydrogen phosphate (KH_2_PO_4_) and di-sodium hydrogen phosphate (Na_2_HPO_4_) were from Kemika (Zagreb, Croatia). All of the reagents were of analytical quality.

### 3.2. Isolation of Sinapine

SI was isolated from kale (*Brassica oleracea*) seeds according to the procedure described by Terpinc et al. [37]. Ground seeds were mixed with boiling water for 5 min, with the mixture shaken at room temperature for 2 h, and then filtered. This water extract was appropriately diluted with carbonate buffer to obtain a neutral pH, and the resulting mixture was filtered through a 0.45-μm membrane filter. SI isolation was carried out by solid phase extraction using Strata-X-CW cartridges (Phenomenex, Torrance, CA, USA), by consecutive passing of methanol, carbonate buffer (pH 7), and then the water extract through the cartridges. SI was captured on the solid phase by the weak cation-exchange mechanism, with the cartridges then washed using water and methanol. The SI fraction was eluted with 4 mL 2% formic acid in 70% methanol (*v*/*v*). The final purity of the isolated product was determined by liquid chromatography using photodiode array detection (LC-DAD, Agilent Technologies, Santa Clara, CA, USA), with its structure confirmed by liquid chromatography–mass spectrometry (LC-MS, Agilent Technologies, Santa Clara, CA, USA and Quatro micro API, Waters, Milford, MA, USA) and nuclear magnetic resonance (NMR) spectroscopy (Agilent Technologies, Santa Clara, CA, USA).

### 3.3. Synthesis of 4-Vinylsyringol

VS was prepared by thermal decarboxylation of SA according to the method of Terpinc et al. [38]. Briefly, SA was dissolved in dimethylformamide, and sodium acetate was added as a catalyst for the decarboxylation. The reaction mixture was heated in an oil bath at 130 °C for 1 h. Milli-Q water was then added, and the product was extracted using diethyl ether. The organic phase was collected and further washed with carbonate buffer at pH 7. After drying with anhydrous Na_2_SO_4_, the organic solvent was removed under reduced pressure, and the precipitate was dissolved in methanol.

The product was passed into 500-mg Strata-X cartridges (Phenomenex, Torrance, CA, USA) that had been previously conditioned with methanol. This was followed by a 50% (*v*/*v*) methanol wash, and the product eluted with 50%, 60%, and 70% (*v*/*v*) methanol, in this order. The less polar compounds that remained were eluted with 100% methanol. The purity of the individual fractions was determined by LC-DAD. In the next step, the eluates that contained more than 95% VS were combined, adequately diluted, and loaded onto Strata-X cartridges previously conditioned with methanol. This was followed by 25% (*v*/*v*) methanol, and elution with 100% methanol. These eluates were filtered through 0.20-μm filters and evaporated to dryness under a vacuum, at 30 °C. The product was weighed and re-dissolved in methanol. The final purity of the synthesised product was determined by LC-DAD. For confirmation of the structure of the synthesised product, LC-MS and NMR analysis were used.

### 3.4. Synthesis of Sinapoyl Esters

The alkyl esters of SA were synthesised by acid catalysed esterification, using the method of Gaspar et al. [39]. SA (1.0 g) was dissolved in 75 mL of the corresponding alcohol (i.e., ethanol, *n*-propanol, or *n*-butanol) containing 1 mL H_2_SO_4_, and the solutions were stirred at room temperature for 5 days. The solvents were partially evaporated under reduced pressure, and the mixtures were then extracted with diethyl ether (3 × 75 mL). The organic phases were combined, washed with 10% Na_2_CO_3_ solution, and dried over anhydrous Na_2_SO_4_. The solvent was evaporated under reduced pressure, and the crude products were purified by recrystallization. The final purities of these synthesised products were determined by LC-DAD, and their structures confirmed by LC-MS and NMR analysis.

### 3.5. Liquid Chromatography with Photodiode Array Detection

The purities of the synthesised/isolated products were determined using LC-DAD (Agilent 1100 binary pump [G1312A], autosampler [G1330B] and a DAD [G]) as follows: the samples (10 μL) were injected onto a reversed-phase C18 column (Gemini; 100 × 2.00 mm; 2.6 µm) that was protected by a guard column (Gemini C18 Security Guard cartridge; 4.0 × 2.0 mm) (Phenomenex, USA). The column thermostat was set at 25 °C. The solvent system used was 0.1% (*v*/*v*) aqueous formic acid (solvent A), and acetonitrile (solvent B). A binary gradient was used at a flow rate of 0.25 mL/min, as follows (as % B in A): 0–4 min, 10% B; 4–40 min, 10%–60% B; 40–41 min, 60%–80% B; 41–47 min, 80% B; 47–50 min, 80%–10% B; 50–60 min, 10% B. The chromatograms were recorded from 240 nm to 650 nm.

### 3.6. Liquid Chromatography–Mass Spectrometry

**Figure 5:**
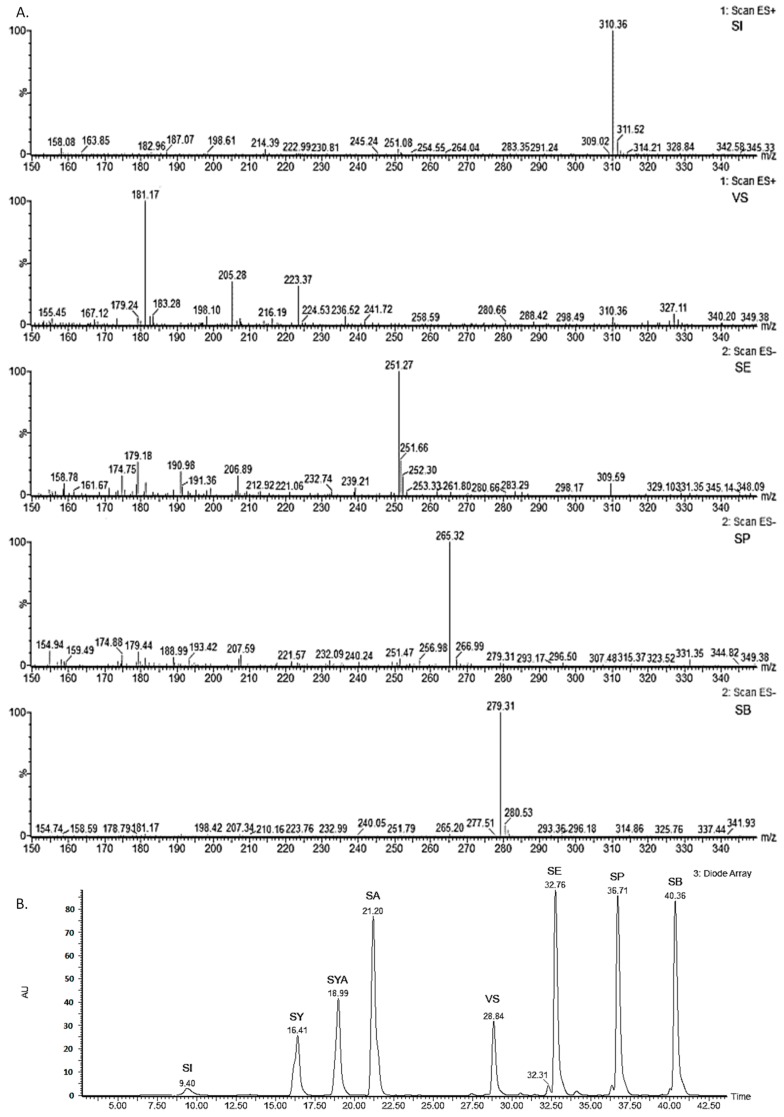
(**A**) Mass spectra of the synthesized/isolated sinapic acid derivatives. ESI+: sinapine (SI), *m*/*z* 310.36; 4-vinylsyringol (VS), *m*/*z* 181.17. ESI−: ethyl sinapate (SE), *m/z* 251.27; propyl sinapate (SP), *m*/*z* 265.32; butyl sinapate (SB), *m*/*z* 279.31; (**B**) Photodiode array detection chromatograms (240–650 nm) of sinapic acid (SA), syringic acid (SY), and syringaldehyde (SYA), and the synthetised/isolated sinapic acid derivatives ethyl sinapate (SE), propyl sinapate (SP), butyl sinapate (SB), sinapine (SI), and 4-vinylsyringol (VS).

The mass spectra of the synthesised/isolated products were recorded using a mass selective detector (Quattro micro API; Waters, Milford, MA, USA) equipped with electrospray ionization (ESI), using a cone voltage of 30 V and a capillary voltage of 3.0 kV for positive/ negative ionisation of the analytes (i.e., ESI+ or ESI− modes). The dry nitrogen for the detector was heated to 300 °C, and the drying gas flow was 400 L/h. The cone gas (nitrogen) flow was 70 L/h. The data were acquired in positive or negative ESI scan modes (in mass range 100–615). The mass spectra for the synthesised and isolated compounds are given in Figure 5.

### 3.7. Nuclear Magnetic Resonance Spectroscopy

Nuclear magnetic resonance spectroscopy was performed on an NMR spectrometer (DD2; Agilent Technologies, Santa Clara, CA, USA) at frequencies of 297.80 MHz (^1^H) and 74.89 MHz (^13^C) at 25 °C. The samples were solubilised in deuterated dimethyl sulphoxide (DMSO-*d*_6_). The chemical shifts (δ) are referenced to the residual solvent signal of DMSO-*d*_6_ and reported in ppm, while the coupling constants (*J*) are given in Hz (Table 2). The ^1^H- and ^13^C-NMR chemical shifts and H–H coupling constants for compounds SI, VS, SE, SP, and SB are given in the Table 2.

### 3.8. DPPH˙ Radical Scavenging Activity

The DPPH˙ radical scavenging for SA and its derivatives was determined according to Brand-Williams et al. [40]. A solution of the DPPH˙ radical in ethanol was added to ethanolic solutions of these compounds (with ethanol alone as the control), to their final concentration in the reaction mixtures of 6.67 × 10^−5^ mol/L. The absorbances of the samples (*A*s^517nm^_(*t*=*x*)_) and the control (*A*c^517nm^_(*t*=0)_) were monitored continuously at 517 nm at regular time intervals (5 s) over 30 min, against ethanol as a blank on a UV-VIS spectrophotometer (model 8453; Hewlett Packard, Waldbronn, Germany). The measurements were performed at room temperature (25 °C), with the analyses carried out from two consecutive runs.

### 3.9. Superoxide Anion Scavenging Activity

Measurements of the superoxide anion scavenging activities of the selected compounds were carried out by the method described by Roback and Gryglewski [41]. All of the reagents were prepared in 0.1 M phosphate buffer, pH 7.4. An aliquot of each compound (with ethanol alone as the control) was mixed with the 150 μM NBT and 468 μM NADH solutions, where the final concentration of the tested compounds was 5 × 10^−3^ mol/L. The reactions were started by addition of the 60 μM PMS solution to the mixture. The absorbances of the samples (*A*s^560nm^_(*t*=*x*)_) and of the controls (*A*c^560nm^_(*t*=*x*)_) were monitored at 560 nm, against a blank sample (without PMS) at 5 s time intervals over a period of 5 min at room temperature. All of these analyses were carried out in duplicate and the results are given as the means.

### 3.10. Antioxidant Activity in the β-Carotene-linoleic Acid Emulsion System

The antioxidant activities of the selected compounds in an aqueous emulsion system of linoleic acid and β-carotene were determined according to Moure et al. [30]. β-Carotene in chloroform (1 mL, 0.2 mg/mL) was mixed with 20 mg of linoleic acid and 200 mg of Tween 20. After evaporation of the chloroform, 50 mL Milli-Q water was added and mixed thoroughly. The selected compounds were added to 2.5 mL of this emulsion to the final concentration of 2 × 10^−4^ mol/L, and then mixed thoroughly. The samples were immediately placed in a water bath at 50 °C and incubated for 2 h. A control was prepared using ethanol instead of the selected compounds. Oxidation of the β-carotene emulsion was monitored spectrophotometrically by recording the absorbance of the samples (*A*s^470nm^_(*t*=*x*)_) and the control (*A*c^470nm^_(*t*=*x*)_) at 470 nm over 2 h, at regular time intervals of 1 min during the first 5 min, and then every 5 min for the remaining time, against a blank consisting of the emulsion without β-carotene. This was carried out at 50 °C on a UV-VIS spectrophotometer (model Varian Cary 100 Bio; Varian Inc., Palo Alto, CA, USA). Duplicate analyses were run for each selected compound.

### 3.11. Lipophilicity Determination

The lipophilicity of the selected SA derivatives was determined using *CPV*, as described by Terpinc et al. [15], and these are given in Table 1. *CPV*s were calculated as the volume ratio between the acetonitrile and aqueous formic acid mobile phases at the moment of elution. A higher *CPV* indicates higher content of acetonitrile in the mobile phase and slower elution of the analyte from the column, and consequently lower polarity of the compound.

## 4. Conclusions

In this investigation a kinetic approach was used in combination with standard fixed endpoint radical scavenging analysis to examine the scavenging activities and reaction mechanisms of sinapic acid and its derivatives against DPPH˙, O_2_˙^−^ and lipid radicals. The results were in accordance with proposed mechanisms of free radical scavenging and differences in reactivity were interpreted as a function of BDE, IP, PA, and ETE molecular descriptors, acid-base properties, steric effects, and the lipophilicity of the selected compounds. On the other hand, it has been demonstrated that the kinetics response of an antioxidant is dependent also on the radical species and the nature of the medium. Accordingly, the important considerations in the selection of the appropriate antioxidant for any kind of system (e.g., food matrix) are the properties of the system itself.

Our results suggest that the measurements of early response parameters (*R*s, *R*_FF_, *R*_B_) can be used to differentiate between antioxidants with comparable fixed endpoint parameters (*I*_DPPH_, *C*_SASA_, *C*_AA_). This combined approach allows more comprehensive characterization of antioxidants compared to the standard methodology which focuses on measurements at the fixed endpoint. In addition, analysis of our results suggests that O_2_˙^−^ scavenging follows ET mechanism which has not yet been classified into any of the mechanistic models in the literature.

## Figures and Tables

**Table 1:** The rates of DPPH˙ radical scavenging (*R*_S_) at 0.1 s, the formazan formation (*R*_FF_) at 10 s, and the β-carotene bleaching (*R*_B_) at 10 s, and the determination coefficients for the kinetics of the DPPH˙ radical scavenging (*r*^2^ (DPPH˙)), the O_2_˙^−^ scavenging (*r*^2^ (O_2_˙^−^)), and the β-carotene bleaching (*r*^2^ (β-carotene)). The data for the DPPH˙ scavenging activity (*I*_DPPH_) estimated at 30 min, the superoxide radical scavenging activity coefficient (*C*_SASA_) estimated at 5 min, and the antioxidant activity coefficient in emulsion (*C*_AA_) estimated at 60 min. Chromatographic partition values (*CPV*) are also given.

Selected Compound	*R*_S_ × 10^−2^ (s^−1^)	*R*_FF_ × 10^−3^ (s^−1^)	*R*_B_ × 10^−4^ (s^−1^)	*r*^2^ (DPPH˙)	*r*^2^ (O_2_^˙−^)	*r*^2^ (β-Carotene)	*I*_DPPH_ (%) ^a^	*C*_SASA_ (%) ^a^	*C*_AA_ (%) ^a^	*CPV*
Sinapic acid (SA)	15.40	1.57	10.54	0.946	0.967	0.978	86 ± 0.4	66 ± 4	29 ± 2	0.46
Sinapine (SI)	0.27	14.21	7.62	0.967	0.996	0.967	23 ± 0.5	0	2 ± 0.5	0.20
Vinyl syringol (VS)	34.83	2.31	7.33	0.967	0.965	0.984	74 ± 0.6	26 ± 1	60 ± 1	0.73
Syringic acid (SY)	1.21	0.92	4.78	0.962	0.953	0.975	80 ± 1	75 ± 0.1	6 ± 1	0.34
Syringaldehyde (SYA)	0.05	6.37	3.37	0.447	0.981	0.968	6 ± 0.6	23 ± 3	1 ± 0.2	0.40
Ethyl sinapate (SE)	46.27	9.88	6.46	0.901	0.988	0.987	69 ± 1	5 ± 1	62 ± 2	0.90
Propyl sinapate (SP)	81.44	ND	2.79	0.969	ND	0.984	67 ± 1	ND	73 ± 1	1.13
Butyl sinapate (SB)	35.93	ND	1.99	0.875	ND	0.980	67 ± 0.8	ND	75 ± 1	1.38

^a^ Means of two replicates ± standard deviation; ND, not determined.

**Table 2:** ^1^H- and ^13^C-NMR chemical shifts (in ppm) and H–H coupling constants (in Hz) for the synthesized/isolated compounds. s, singlet; b, broad; d, doublet; m, multiplet.

Compound	^1^H- and ^13^C-NMR Spectroscopic Data
Sinapine (SI)	δ_H_ (297.80 MHz, DMSO-*d*_6_): 7.61 (d, *J =* 15.9, 1H, –CH=), 7.03 (s, 2H, Ar–H), 6.55 (d, *J =* 15.9, 1H, =CH–), 4.58 (s, 2H, –OCH_2_), 3.80 (s, 6H, –OCH_3_), 3.72 (m, 2H, CH_2_) 3.17 (s, 9H, –NCH_3_); δ_C_ (74.89 MHz, DMSO-*d*_6_): 165.93 (C=O), 148.07 (C(3), C(5)), 146.25 (C(β)), 138.82 (C–OH), 124.05 (C(1)), 113.97 (C(α)), 106.45 (C(2), C(6)), 63.97 (CH_2_), 57.65 (CH_2_), 56.13 (2× OCH_3_), 52.99 (N(CH_3_)_3_)
4-Vinylsyringol (VS)	δ_H_ (297.80 MHz, DMSO-*d*_6_): 8.44 (s, 1H, OH), 6.73 (s, 2H, Ar–H), 6.60 (d, *J =* 17.6, 10.8, 1H, –CH=), 5.67 (d, *J =* 17.6, 1.1, 1H, =CH_2_), 5.08 (d, *J =* 10.8, 1.1, 1H, =CH_2_), 3.77 (s, 6H, –OCH_3_); δ_C_ (74.89 MHz, DMSO-*d*_6_): C(6)), 147.97 (C(3), C(5)), 136.94 (C–OH), 135.73 (=CH), 127.66 (C(1)), 111.34 (=CH_2_), 103.77 (C(2), C(6)), 55.94 (2× OCH_3_)
Ethyl sinapate (SE)	δ_H_ (297.80 MHz, DMSO-*d*_6_): 8.94 (s, 1H, OH), 7.55 (d, *J =* 15.9, 1H, –CH=), 7.03 (s, 2H, Ar–H), 6.53 (d, *J =* 15.9, 1H, =CH–), 4.16 (q, *J =* 7.1, 2H, –OCH_2_), 3.80 (s, 6H, –OCH_3_), 1.25 (t, *J* = 7.1, 3H, CH_3_); δ_C_ (74.89 MHz, DMSO-*d*_6_): 166.55 (C=O), 147.98 (C(3), C(5)), 145.19 (C(β)), 138.23 (C–OH), 124.36 (C(1)), 114.96 (C(*α*)), 106.19 (C(2), C(6)), 59.66 (CH_2_), 56.06 (2× OCH_3_), 14.23 (CH_3_)
Propyl sinapate (SP)	δ_H_ (297.80 MHz, DMSO-*d*_6_): 8.93 (s, 1H, OH), 7.55 (d, *J =* 15.9, 1H, –CH=), 7.03 (s, 2H, Ar–H), 6.53 (d, *J =* 15.9, 1H, =CH–), 4.08 (t, *J =* 6.6, 2H, –OCH_2_), 3.80 (s, 6H, –OCH_3_), 1.65 (t, *J =* 7.4, 6.6, 2H, CH_2_), 0.93 (t, *J =* 7.4, 3H, CH_3_); δ_C_ (74.89 MHz, DMSO-*d*_6_): 166.65 (C=O), 147.98 (C(3), C(5)), 145.22 (C(β)), 138.23 (C–OH), 124.36 (C(1)), 114.91 (C(α)), 106.20 (C(2), C(6)), 65.18 (CH_2_), 56.06 (2× OCH_3_), 21.66 (CH_2_), 10.33(CH_3_)
Butyl sinapate (SB)	δ_H_ (297.80 MHz, DMSO-*d*_6_): 8.94 (s, 1H, OH), 7.54 (d, *J =* 15.9, 1H, –CH=), 7.03 (s, 2H, Ar–H), 6.53 (d, *J =* 15.9, 1H, =CH–), 4.12 (t, *J =* 6.6, 2H, –OCH_2_), 3.80 (s, 6H, –OCH_3_), 1.61 (m, 2H, CH_2_), 1.38 (m, 2H, CH_2_), 0.92 (t, *J =* 7.3, 3H, CH_3_); δ_C_ (74.89 MHz, DMSO-*d*_6_): 166.65 (C=O), 147.98 (C(3), C(5)), 145.21 (C(β)), 138.23 (C–OH), 124.36 (C(1)), 114.92 (C(α)), 106.20 (C(2), C(6)), 63.38 (CH_2_), 56.06 (2× OCH_3_), 30.33 (CH_2_), 18.67 (CH_2_), 13.57 (CH_3_)
